# Quantitation of relationship and development of nutrient prediction with vibrational molecular structure spectral profiles of feedstocks and co-products from canola bio-oil processing

**DOI:** 10.5713/ab.22.0076

**Published:** 2022-06-30

**Authors:** Alessandra M. R. C. B. de Oliveira, Peiqiang Yu

**Affiliations:** 1 Department of Animal and Poultry Science, College of Agriculture and Bioresources, University of Saskatchewan, Saskatoon, SK, S7N 5A8, Canada

**Keywords:** Canola Bio-oil Processing, Feedstock and Co-products, Interactive Relationship, Molecular Structures, Nutrient Utilization and Availability

## Abstract

**Objective:**

This program aimed to reveal the association of feed intrinsic molecular structure with nutrient supply to animals from canola feedstocks and co-products from bio-oil processing. The special objective of this study was to quantify the relationship between molecular spectral feature and nutrient availability and develop nutrient prediction equation with vibrational molecular structure spectral profiles.

**Methods:**

The samples of feedstock (canola oil seeds) and co-products (meals and pellets) from different bio-oil processing plants in Canada (CA) and China (CH) were submitted to this molecular spectroscopic technique and their protein and carbohydrate related molecular spectral features were associated with the nutritional results obtained through the conventional methods of analyses for chemical and nutrient profiles, rumen degradable and intestinal digestible parameters.

**Results:**

The results showed that the spectral structural carbohydrates spectral peak area (ca. 1,487.8 to 1,190.8 cm^−1^) was the carbohydrate structure that was most significant when related to various carbohydrate parameters of canola meals (p<0.05, r>0.50). And spectral total carbohydrate area (ca. 1,198.5 to 934.3 cm^−1^) was most significant when studying the various carbohydrate parameters of canola seeds (p<0.05, r>0.50). The spectral amide structures (ca. 1,721.2 to 1,480.1 cm^−1^) were related to a few chemical and nutrient profiles, Cornell Net Carbohydrate and Protein System (CNCPS) fractions, truly absorbable nutrient supply based on the Dutch protein system (DVE/OEB), and NRC systems, and intestinal in vitro protein-related parameters in co-products (canola meals). Besides the spectral amide structures, α-helix height (ca. 1,650.8 to 1,643.1 cm^−1^) and β-sheet height (ca. 1,633.4 to 1,625.7 cm^−1^), and the ratio between them have shown to be related to many protein-related parameters in feedstock (canola oil seeds). Multi-regression analysis resulted in moderate to high R^2^ values for some protein related equations for feedstock (canola seeds). Protein related equations for canola meals and carbohydrate related equations for canola meals and seeds resulted in weak R^2^ and low p values (p<0.05).

**Conclusion:**

In conclusion, the attenuated total reflectance Fourier transform infrared spectroscopy vibrational molecular spectroscopy can be a useful resource to predict carbohydrate and protein-relates nutritional aspects of canola seeds and meals.

## INTRODUCTION

The dairy production system, especially in Canada, uses canola meal, rather than the seeds a source of protein because canola seeds are largely crushed for its oil content generating the meal as a co-product. The literature indicates that changes in temperature and time of harvesting can alter the chemical composition of canola seeds and different processing methods can alter the composition of canola meals [1]. Furthermore, the chemical composition of feedstuffs is indispensable in animal nutrition for feeds account for around 60–75% of the costs in animal production.

Wet laboratory analyses methods and *in vivo* studies require intensive labor, high amounts of samples and long hours. And each day the industry brings forth a new variety of plant, a different method of processing etc. and all can affect the final product that is consumed by the animals, therefore determining the chemical composition is required and the faster this information can be obtained, the faster the industry can improve, and better animal performance and increased profits can be observed. Therefore, a fast method of analysis for canola seeds and meals would be helpful in the dairy industry, saving time in analysis and money in manipulating diets that are taking into consideration the real specific characteristics of the ingredients being used.

As an alternative to time consuming wet laboratory analysis, different infrared spectroscopy methods have gained space in animal nutrition [2,3]. Spectroscopy is being used because it studies matters though its interaction with light quickly and without damaging the sample. The attenuated total reflectance Fourier transform infrared spectroscopy (ATR-FTIR) analyzes the interaction of matter with infrared light on the mid-infrared region (ca. 4,000 to 800 cm^−1^) in a quick and non-destructive manner [2]. This is different from the wet analyses that use chemicals and procedures that can damage structures and alter the composition and digestibility of feeds [4–6].

ATR-FTIR can help us learn not only about the composition of an ingredient but also what kind of response that ingredient has when fed to a ruminant. Therefore, to understand how the intrinsic molecular structures of canola seeds and meals relate to the chemical composition, energy profile, degradability, and digestibility in the gastrointestinal tract of dairy cows is an advantage for the industry and was the aim of this study.

This large program aimed to reveal the association of feed intrinsic molecular structure with nutrient supply to animals from canola feedstocks and co-products from bio-oil processing. The special objective of this current study was to quantify the relationship between molecular spectral feature and nutrient availability and develop nutrient prediction equation with vibrational molecular structure spectral profiles.

## MATERIALS AND METHODS

The University of Saskatchewan Animal Care Committee approved the animal trial under the Animal Use Protocol No. 19910012 and animals were cared for and handled in accordance with the Canadian Council of Animal Care (CCAC, 1993) regulations.

### Sampling

Samples from canola bio-oil processing plants in Canada and China were collected by the Canola Council of Canada. Five different companies in Canada provided samples from seeds used and meals produces in five different batches. As well as five different companies in China provided samples from the seeds and meals from five different batches. Each company’s quality control laboratory provided the samples that were later analyzed at the University of Saskatchewan in Canada.

### Chemical analysis, degradation, and intestinal digestion and nutrient supply

Chemical analyses were followed the AOAC official methods of analysis [7]; energy values were determined using a chemical summary approach in NRC [8,9]; protein and carbohydrate fractions were carried out using CNCPS 6.5 system [10,11]; the *in situ* study required the rumen incubation of 7 g samples at 0, 2, 4, 8, 12, 24, and 48 h in four Holstein cows followed the animal care guidelines and approved by the ethics committee of the University of Saskatchewan; the *in vitro* study followed the three-step procedure by Calsamiglia and Stern [12]. The true nutrient supply was studied using NRC [8,9] and the Dutch protein system (DVE/OEB) [13,14]. The spectral analysis was carried out by using the ATR-FTIR technique to study carbohydrate and protein-related molecular structures. All procedures and analyses were reported in detail in the previous studies.

### Statistical analysis

To study the relationship between the various spectral features to the chemical and energy profiles, and rumen and intestinal availability and digestibility, the data of interest were analyzed using the procedure CORR on SAS 9.4 (SAS Institute, USA).

The procedure REG on SAS 9.4 (SAS Institute, USA) was used for the multi-regression analysis to create prediction equations based on the data collected during this study. Only the significant model equations are represented here (R^2^> 0.60).

## RESULTS AND DISCUSSION

Relationship study on carbohydrate-related spectral features and chemical and nutrient profiles and rumen degradation and intestinal digestion

In a correlation analysis, there is a linear relationship between the variables analyzed (p<0.05), and the r value will determine the strength of this relationship, where r = 1 or −1, is a perfect relationship; r = 0.8 or −0.8, indicate a strong relationship; r = 0.6 or −0.6, indicate a moderate relationship; r = 0, indicates absence of linear relationship [15]. Represented from Table 1 to 9 are the correlation between carbohydrate-related molecular structures and variables from chemical and energy profiles, Cornell Net Carbohydrate and Protein System (CNCPS) fractions, NRC, and DVE/OEB systems that showed significance (p<0.05).

The structural carbohydrates spectral peak area (STCA) is the molecular structure that seems to have linear relationships with many of the characteristics of canola meals studied. It is related to the contents of cellulose and lignin (Table 1), digestible fiber fractions (Table 3), effective degradability of protein and microbial protein (MP) synthesized in the rumen based on energy (Table 5), total digestible neutral detergent fiber (tdNDF) and feed milk value (Table 6), and with endogenous crude protein (ECP) and ECP truly absorbed in the small intestine (AECP) (Table 8). While other structures were also related, STCA was related to at least one aspect of each studied profile or system.

The total carbohydrate area (TCA) is the carbohydrate-related structure that was found to be related to many characteristics of canola seeds in this study. It is related to the sugar content (Table 2), rumen degradable and undegradable fractions of water-soluble carbohydrates (RDCA4 and RUCA4) (Table 4), and to ECP and AECP (Table 9). The cellulosic compounds area was linearly related to neutral detergent aspects (NDF and tdNDF) (Tables 2 and 7). None of the carbohydrate molecular structures studied on this project appeared to have a linear relationship with any of the DVE/OEB system variables for canola seeds (p>0.05, values not represented).

Several studies (Theodoridou et al [5]; Chen et al [3]; Ban et al [2]) rereported the relationship between spectral feature collected from different spectroscopic techniques DRIFT, Synchrotron IMS, ATR-FTIR molecular spectroscopy and nutrition. However, there was no systematic relationship study in canola seeds and meal in a large scale. This current study shows that it is possible to relate many feed characteristics and ruminal and intestinal responses of canola seeds and meals fed to dairy cows from their carbohydrate-related spectral profiles revealed through molecular analysis using the ATR/FTIR technology.

### Relationship study on protein-related spectral features and chemical and nutrient profiles and rumen degradation and intestinal digestion of canola seeds and meals

The linear relationship study between protein structures revealed through the ATR/FTIR technique and chemical characteristics of canola meals and seeds that were significant are presented from Table 10 to 19. Many variables from canola meals and seeds showed to be related with amides areas and heights in the present study. Weak correlations are not represented on these tables.

Amides peak area and amides height are related to the soluble crude protein content of both canola meals and seeds (Tables 10, 11), but stronger relationships were observed on canola seeds (r = 0.64, p<0.001, for peak area; r = 0.62, p<0.001, for amide height) (Table 11). Slowly degradable protein fractions (PB2 and RDPB2) of canola meals are negatively related to Amide area, and moderately degradable protein (PB1) is also negatively related to amide area ratio and amide height in canola meals (Table 12). Only the truly digested protein in the small intestine (DVE) (r = 0.57, p = 0.026) and estimated milk production (DVE FMV) (r = 0.56, p = 0.028) of canola meals seemed to be related to the height of amide II (Table 14).

Strong relationships can be observed between many protein structures and soluble protein fractions (PA2, RDPA2, and RUPA2), moderately degradable fractions (PB1, RDPB1, and RUPB1), unavailable protein (PC), total protein (TP), and total degradable (Total RDP) and undegradable protein (Total RUP) (Table 13) of canola seeds. Residue at 0 h and the soluble fraction of canola seeds are related to peak area, amide II area, amide areas ratio, amide I and II heights, and β-sheet height (Table 15).

Different fractions of the *in vitro* digestibility showed relationships in canola seeds and meals. The intestinal digestibility of proteins (IDP) of canola meals was related to the height of Amide II (r = 0.63, and p = 0.012) (Table 16). While both the digestibility of bypass dry matter (dBDM) (r = 0.67, p = 0.007) and the intestinally absorbable feed protein (IADP) (r = 0.65, p = 0.009) were related to the α-helix:β-sheet ratio (Table 17). Amide II height was related to MP on canola meals (r = 0.53, p = 0.043) (Table 18) and to endogenous crude protein (ECP) (r = 0.61, p = 0.016) and AECP (r = 0.60, p = 0.019) on canola seeds (Table 19). On canola seeds, AECP was also related to α-helix (r = 0.63, p = 0.013) and to α-helix:β-sheet ratio (r = 0.88, p<0.001), similarly ECP was also related to α-helix (r = 0.63, p = 0.012) and to α-helix:β-sheet ratio (r = 0.84, p<0.001) (Table 19).

Theodoridou and Yu [5,6] studied the correlation of canola meals and presscake to protein structures and they also found that amide I and II areas and their ratio were related to NDIP (r = 0.95, p = 0.051), PB1 (r = −0.76, p = 0.244), PB2 (r = 0.82, p = 0.188), IDP (r = 0.89, p = 0.107), MP (r = 0.99, p = 0.006). The high r values that they obtained show a tendency for a strong relationship, but the high P values indicate that a higher sample size is necessary to confirm those relationships [16]. Similar to our results, Huang [17] found relationships between the amide I and II areas, heights and their ratios and CP, soluble crude protein, PA2, PB1, tdCP, S, D, and TDP in pelleted canola meals (r>0.76 and p<0.05), but did not find correlation between α-helix height, β-sheet height, and α-helix: β-sheet ratio and any protein parameters of canola meals.

These results, along with ours, indicate that the various processes for oil extraction, desolventizing of the meals, and pelleting may affect the protein structures of the meals differently, even if the companies use similar processes. Or simply, they indicate that repeating the study with higher sample sizes would improve the results and give us a clearer understanding of the correlations between protein structures and the characteristics of canola meals. Although based on our results it seems to be easier to relate protein spectral structures with the protein structures of canola seeds, because more frequent and stronger relationships could be observed for the seeds than for the meals, more repetition could only help to support the results presented here.

### Prediction of nutrient supply and rumen and intestinal digestion of canola seeds and meals using unique molecular spectral features

Multiple regression analysis is used to verify the strength of the relationship between a dependent variable and several predictor variables, quantifying and statistically eliminating the effect of other predictors [18]. In our study, carbohydrate and protein-related spectral structures were used to predict chemical, degradable, and digestible characteristics of canola meals and seeds. However, the most significant prediction equations were relating protein-related structures to ruminal degradability and intestinal digestibility aspects of canola seeds (Table 20). The area of Amide I along with either the heights of α-helix or β-sheet or their ratio seem to be good predictors of rumen degradable and undegradable soluble and moderately soluble protein fractions (PA2, RDPA2, RDPB1, RUPA2, and RUPB1), and of the total rumen undegraded protein (Total RUP) in canola seeds (p<0.05, R^2^≥0.65). These results are important because in ruminants there is an extensive use of nitrogen compounds by the ruminal microbiota and that affects the quantity and quality of protein available for digestion in the small intestine. Being able to predict with more confidence how much of the protein in the canola seeds will be available for the animal to use, is extremely helpful in animal nutrition.

Although a low R^2^ means variation in the results, this variability shows a trend behavior of the variables studied. All the prediction equations for canola meals using protein-related structures showed low R^2^ but they also showed very low p-values (Table 20). This indicates that even not being too precise, a trend is observed between those variables, and α-helix height is the protein-related structure that appears to be a good predictor for many energy-related variables. Crude protein and total digestible crude protein (TDCP) also showed a trend to be predicted by the α-helix height and amide II height. A similar response can be observed between some carbohydrate-related structures and some aspects of canola meals and seeds (Table 21). These results clearly show a pattern and further analysis with more data would likely increase the R^2^ values and give more assurance to the users of these equations.

## CONCLUSION

The correlation study between carbohydrate spectral features and canola meals and seeds showed that STCA commonly appear to be related to canola meals and TCA to canola seeds features. And the correlation between protein spectral features and canola meals and seeds aspects showed strong relationships with the amide region of both seeds and meals, but more and stronger relationships were observed on canola seeds. These results indicate that the carbohydrate and protein structures obtained with FTIR-ATR have been proven to be related to aspects of canola seeds and meals’ chemical and nutrient profiles, as well as rumen degradable and intestinal digestibility characteristics. Also, the multi-regression analysis of canola meals and seeds and carbohydrate and protein-related molecular structures showed trends between protein-related structures for the canola meals equations (p≤0.004 and R^2^≥0.23). However, high R^2^ (>0.64) and low p values (≤0.002) observed for canola seeds using protein molecular structures indicate that a higher trust can be put onto those equations.

## Figures and Tables

**Table 1:** Correlation between FTIR carbohydrate structures and the carbohydrate portions of the chemical profile of canola meals

Items	TC1H		TC2H		TC3H		CECH		STCA	
				
	r	p-value	r	p-value	r	p-value	r	p-value	r	p-value
NDF (% DM)			−0.57	0.027	−0.52	0.049				
Hemicellulose (% DM)			−0.54	0.036						
Cellulose (% DM)							0.55	0.035	0.64	0.011
ADL (% NDF)									−0.60	0.018

FTIR, Fourier transform infrared; TCxH, total carbohydrate peak height; CECH, cellulosic compounds peak height; STCA, structural carbohydrate area; 1, 2, 3 and 4: correspond to the different peaks; r, correlation coefficient using Spearman; NDF, neutral detergent fiber; DM, dry matter; ADF, acid detergent fiber; ADL, acid detergent lignin.

Missing values had p>0.05.

**Table 2:** Correlation between FTIR carbohydrate structures and the carbohydrate portions of the chemical profile of canola seeds

Items	TC2H		STC1H		CECA		TCA	
			
	r	p-value	r	p-value	r	p-value	r	p-value
NDF (% DM)	−0.56	0.031			−0.53	0.044		
ADL (% DM)			0.66	0.008				
NFC (% CHO)								
Sugar (% DM)	0.56	0.030	−0.58	0.022			0.69	0.004

FTIR, Fourier transform infrared; TCxH, total carbohydrate peak height; STC1H, structural carbohydrate area; 1 peaks; H, peak height; CECA, cellulosic compounds area; TCA, total carbohydrate area; r, correlation coefficient using Spearman; NDF, neutral detergent fiber; DM, dry matter; ADL, acid detergent lignin; NFC, non-fibre carbohydrates.

Missing values had p>0.05.

**Table 3:** Correlation between FTIR carbohydrate structures and the carbohydrate portions of the CNCPS system of canola meals

Items	TC2H		CECH		STC1H		STC2H		CECA		TCA		STCA	
						
	r	p-value	r	p-value	r	p-value	r	p-value	r	p-value	r	p-value	r	p-value
CB3 (%CHO)	−0.56	0.029			0.60	0.018			0.55	0.032				
RDCB3	−0.52	0.045			0.54	0.037			0.53	0.041			0.56	0.028
Total RDC			0.55	0.033			0.68	0.005						
RUCA4														
RUCB3	−0.52	0.045			0.54	0.037			0.53	0.041			0.56	0.028
RUCC											−0.52	0.046		

FTIR, Fourier transform infrared; CNCPS, Cornell Net Carbohydrate and Protein System; TCxH, total carbohydrate peak height; CECH, cellulosic compounds peak height; STCxH, structural carbohydrate area; 1, 2: correspond to the different peaks; H, peak height; CECA, cellulosic compounds area; TCA, total carbohydrate area; STCA, structural carbohydrate area; 1, 2, 3 and 4: correspond to the different peaks; r, correlation coefficient using Spearman; CB3, ruminaly degradable carbohydrate fraction of available NDF; RDCB3, ruminally degradable digestible fiber; Total RDC, total ruminally degradable carbohydrates; RUCA4, ruminally undegradable water soluble carbohydrates; RUCB3, ruminally undegradable digestible fiber; RUCC, ruminally indigestible fiber.

Missing values had p>0.05.

**Table 4:** Correlation between FTIR carbohydrate structures and the carbohydrate portions of the CNCPS system of canola seeds

Items	TC2H		TC3H		STC1H		TCA	
			
	r	p-value	r	p-value	r	p-value	r	p-value
CB2 (% CHO)					0.56	0.030		
CC (% CHO)					0.65	0.008		
RDCA4	0.56	0.030			−0.58	0.022	0.69	0.004
RUCA4	0.56	0.029	0.52	0.048	−0.57	0.027	0.69	0.004

FTIR, Fourier transform infrared; CNCPS, Cornell Net Carbohydrate and Protein System; TCxH: total carbohydrate peak height; STC1H, structural carbohydrate area; 1 peaks; H, peak height; TCA, total carbohydrate area; r, correlation coefficient using Spearman; CB2, soluble fiber; CC, unavailable fiber; RDCA4, ruminally degradable water soluble carbohydrates; RUCA4, ruminally undegradable water soluble carbohydrates.

Missing values had p>0.05.

**Table 5:** Correlation between FTIR carbohydrate structures and the DVE/OEB system for canola meals

Items	STCA	

	r	p-value
EDCP	0.62	0.014
MREE	0.62	0.014

FTIR, Fourier transform infrared; DVE/OEB, the Dutch protein system; STCA, structural carbohydrate spectral area; EDCP, effective degradability of CP; MREE, microbial protein synthesized in the rumen based on the energy available; r, correlation coefficient using Spearman.

**Table 6:** Correlation between FTIR carbohydrate structures and the energy profile of canola meals

Items	TC3H		STCA	
	
	r	p-value	r	p-value
tdNDF			0.65	0.008
Estimated milk	0.55	0.034		

FTIR, Fourier transform infrared; TC, total carbohydrate; STC, structural carbohydrate area; H, peak height; A, peak area; r, correlation coefficient using Spearman; tdNDF, total digestible neutral detergent fiber; Estimated milk, estimated milk production based on energy.

Missing values had p>0.05.

**Table 7:** Correlation between FTIR carbohydrate structures and the energy profile of canola seeds

Items	CECA	

	r	p-value
tdNDF	−0.54	0.038

FTIR, Fourier transform infrared; CEC, cellulosic compound area; A, peak area; r, correlation coefficient using Spearman; tdNDF, total digestible neutral detergent fiber.

**Table 8:** Correlation between FTIR carbohydrate structures and the NRC system for canola meals

Items	STC2H		STC3H		STC4H		STCA	
			
	r	p-value	r	p-value	r	p-value	r	p-value
AECP	−0.58	0.024	−0.80	<0.001	−0.75	0.001	−0.86	<0.001
ECP	−0.58	0.025	−0.80	<0.001	−0.75	0.001	−0.85	<0.001

FTIR, Fourier transform infrared; NRC, National Research Council; STC, structural carbohydrate area; 1, 2, 3 and 4: correspond to the different peaks; H, peak height; A, peak area; r, correlation coefficient using Spearman; AECP, truly absorbed ECP in the small intestine; ECP, endogenous crude protein in the small intestine.

**Table 9:** Correlation between FTIR carbohydrate structures and the NRC system for canola seeds

Items	TC2H		TC3H		TC4H		CECH		STC1H		STC4H		TCA	
						
	r	p-value	r	p-value	r	p-value	r	p-value	r	p-value	r	p-value	r	p-value
AECP	0.57	0.027	0.73	0.002	0.56	0.029	0.72	0.002	−0.96	<0.001	0.55	0.032	0.79	<0.001
ECP	0.60	0.019	0.74	0.002	0.57	0.026	0.75	0.001	−0.96	<0.001	0.58	0.024	0.81	<0.001

FTIR, Fourier transform infrared; TC, total carbohydrate; CEC, cellulosic compounds; STC, structural carbohydrate area; 1, 2, 3 and 4: correspond to the different peaks; H, peak height; A, peak area; r, correlation coefficient using Spearman; AECP, truly absorbed ECP in the small intestine; ECP, endogenous crude protein in the small intestine.

**Table 10:** Correlation between FTIR protein structures and the protein portions of the chemical profile of canola meals

Item	SCP (% CP)		NDIP (% CP)		NDIP (% DM)	
		
	r	p-value	r	p-value	r	p-value
Peak area	0.51	<0.001	−0.52	<0.001	−0.54	<0.001
Height	0.53	<0.001				

FTIR, Fourier transform infrared; SCP, soluble crude protein; NDIP, neutral detergent-insoluble crude protein; Peak area, Amide I and II peak area; Height, ratios of amide I and II heights; r, correlation coefficient using spearman; DM, dry matter; CP, crude protein.

**Table 11:** Correlation study between FTIR protein structures and the protein portions of the chemical profile of canola seeds

Item	SCP (% CP)	

	r	p-value
Peak area	0.64	<0.001
Area ratio	−0.52	<0.001
Height	0.62	<0.001

FTIR, Fourier transform infrared; CP, crude protein; SCP, soluble crude protein; Peak area, Amide I and II peak area; Area ratio, ratios of amide I and amide II areas; Height, ratios of amide I and II heights.

**Table 12:** Correlation study between FTIR protein structures and the protein portions of the CNCPS system profile of canola meals

Item	PB2 (% CP)		PB1 (% CP)		PB1 (% TP)		PB2 (% TP)		RDPB2 (% DM)	
				
	r	p-value	r	p-value	r	p-value	r	p-value	r	p-value
AII	−0.52	0.046					−0.55	0.034	−0.52	0.049
Area ratio			−0.57	0.025	−0.57	0.028				
Height					−0.53	0.041				

FTIR, Fourier transform infrared; CNCPS, Cornell Net Carbohydrate and Protein System; PB1, moderately degradable protein; CP, crude protein; PB2, slowly degradable protein; TP, true protein; RD, rumen degradable; AII, Amide II area; Area ratio, ratios of amide I and amide II areas; Height, ratios of amide I and II heights; r, correlation coefficient using Spearman.

**Table 13:** Correlation study between FTIR protein structures and the protein portions of the CNCPS profile of canola seeds

Items	PA2		PC		PB1		TP		RDPA2		RDPB1	
					
	r	p-value	r	p-value	R	p-value	r	p-value	r	p-value	r	p-value
Peak area	0.74	0.002			−0.63	0.012			0.75	0.001	−0.64	0.010
AI	0.76	0.001			−0.64	0.010			0.76	0.001	−0.65	0.009
AII	0.56	0.030							0.59	0.021		
AIH	0.65	0.008			−0.56	0.030			0.67	0.006	−0.57	0.026
AIIH	0.57	0.028							0.60	0.019	−0.52	0.047
Height			0.58	0.023			−0.58	0.023				
Alpha	0.74	0.002			−0.69	0.004			0.76	0.001	−0.68	0.005
Beta	0.55	0.034							0.55	0.032		
Ratio												

	**Total RDP**		**RUPA2**		**RUPB1**		**RUPC**		**Total RUP**			
						
	**r**	**p-value**	**r**	**p-value**	**r**	**p-value**	**r**	**p-value**	**r**	**p- value**		

Peak area	0.76	0.001	0.75	0.001	−0.64	0.001			−0.74	0.002		
AI	0.75	0.001	0.76	0.001	−0.65	0.001			−0.78	<0.001		
AII	0.60	0.017	0.59	0.021					−0.59	0.022		
AIH	0.69	0.004	0.67	0.006	−0.57	0.026			−0.65	0.009		
AIIH	0.64	0.010	0.60	0.019	−0.52	0.047	−0.53	0.041	−0.59	0.021		
Height							0.56	0.031				
Alpha	0.79	<0.001	0.76	0.001	−0.68	0.005			−0.69	0.005		
Beta	0.56	0.030	0.55	0.032					−0.56	0.029		
Ratio	0.57	0.027										

FTIR, Fourier transform infrared; CNCPS, Cornell Net Carbohydrate and Protein System; PA2, soluble true protein; PC, unavailable crude protein; PB1, moderately degradable protein; TP, true protein; PB2, slowly degradable protein; Total RDP, total rumen degradable protein; RD, rumen degraded; RU, rumen undegraded; Total RUP, total rumen undegradable protein; Peak area, Amide I and II peak area; AI, Amide I area; AII, Amide II area; Area ratio, ratios of amide I and amide II areas; AIH, Amide I height; AIIH, Amide II height; Height, ratios of amide I and II heights; Alpha, α-helix height; Beta, β-sheet height; Ratio, ratio of α-helix: β-sheet; r, correlation coefficient using Spearman.

**Table 14:** Correlation study between FTIR protein structures of Canola meals and the DVE/OEB system

Items	DVE		DVE FMV	
	
	r	p-value	r	p-value
AIIH	0.57	0.026	0.56	0.028

FTIR, Fourier transform infrared; DVE, truly digested protein in the small intestine; DVE FMV, estimated milk production based on the DVE system in kg milk/kg DM feed; AIIH, Amide II height; r, correlation coefficient using Spearman.

**Table 15:** Correlation study between FTIR protein structures and the protein portions of the in situ rumen incubation of canola seeds

Items	Kd (%/h)		Fr (%)		Residue 0 h (%)		S (%)		U (%)		BCP (%)	
					
	r	p-value	r	p-value	r	p-value	r	p-value	r	p-value	r	p-value
Peak area					−0.52	0.046	0.52	0.046				
AII					−0.65	0.008	0.65	0.008				
Area ratio	0.60	0.019			0.66	0.007	−0.66	0.007	0.61	0.017		
AIH					−0.60	0.018	0.60	0.018				
AIIH					−0.58	0.023	0.58	0.023				
Beta					−0.54	0.039	0.54	0.039				
Ratio	−0.76	0.001	−0.60	0.019					−0.65	0.009	0.52	0.046

FTIR, Fourier transform infrared; Kd, the degradation rate of D fraction; Fr, fermentation rate; Residue 0 h: CP residue at 0 h of rumen incubation; S, soluble fraction; U, rumen undegradable fraction; BCP, Bypass CP; Peak area, Amide I and II peak area; AII, Amide II area; Area ratio, ratios of amide I and amide II areas; AIH, Amide I height; AIIH, Amide II height; Alpha, α-helix height; Beta, β-sheet height; Ratio, ratio of α-helix: β-sheet; r, correlation coefficient using Spearman.

**Table 16:** Correlation study between FTIR protein structures and the protein portions of the in vitro of canola meals

Item	IDP (%RUP)	

	r	p-value
AIIH	0.63	0.012

FTIR, Fourier transform infrared; AIIH, amide II height; IDP, intestinal digestibility of protein; r, correlation coefficient using Spearman.

**Table 17:** Correlation study between FTIR protein structures and the protein portions of the in vitro of canola seeds

Item	dBDM (%)		IADP (g/kg DM)	
	
	r	p-value	r	p-value
Ratio	0.67	0.007	0.65	0.009

FTIR, Fourier transform infrared; dBDM, digestibility of bypass dry matter; IADP, intestinally absorbable feed protein; Ratio, ratio of α-helix: β-sheet; r, correlation coefficient using Spearman.

**Table 18:** Correlation study between FTIR protein structures of canola meals and the NRC model

Item	MP	

	r	p-value
AIIH	0.53	0.043

FTIR, Fourier transform infrared; NRC, National Research Council; MP, metabolizable protein; AIIH, Amide II height; r, correlation coefficient using Spearman.

**Table 19:** Correlation study between FTIR protein structures of Canola seeds and the NRC model

Items	AECP		ECP	
	
	r	p-value	r	p-value
AII	0.54	0.040	0.55	0.033
AIIH	0.60	0.019	0.61	0.016
Alpha	0.63	0.013	0.63	0.012
Ratio	0.88	<0.001	0.84	<0.001

FTIR, Fourier transform infrared; NRC, National Research Council; AECP, truly absorbed ECP in the small intestine; ECP, endogenous protein in the small intestine; Peak area, Amide I and II peak area; AII, Amide II area; AIH, Amide I height; AIIH, Amide II height; Alpha, α-helix height; Ratio, ratio of α-helix: β-sheet; r, correlation coefficient using Spearman.

**Table 20:** Best model variables selection in multi-regression analysis to predict Canola protein parameters from FTIR protein structures

Variables (Y)	Prediction equation model: Y = a+b1×X1+b2×X2 …	R2	RSD	p-value
Canola seeds				
PA2 (% CP)	Y = 33.36+5.24×AI−367.58×Ratio	0.72	3.36	<0.001
RDPA2 (% DM)	Y = 5.88+0.83×AI−59.31×Alpha	0.68	0.56	0.001
RDPB1 (% DM)	Y = 4.44–0.47×AI+34.84×Beta	0.64	0.33	0.002
RUPA2 (% DM)	Y = 2.35 + 0.33×AI−23.72×Beta	0.68	0.23	0.001
RUPB1 (% DM)	Y = 6.70 + 0.70×AI+52.15×Beta	0.65	0.50	0.002
Total RUP (% DM)	Y = 12.59–0.40×AI+25.35×Beta	0.75	0.25	<0.001
AECP (% DM)	Y = 3.96–0.15×Area+0.76×Ratio	0.82	0.03	<0.001
ECP (% DM)	Y = 9.97–0.41×Area+1.90×Ratio	0.81	0.07	<0.001
Canola meals				
CP (% DM)	Y = 38.11–15.61×A2H+20.90×Alpha	0.23	0.89	0.004
TD (% CP)	Y = 37.09–17.80×A2H+22.43×Alpha	0.24	0.90	0.004
TDN_1x_	Y = 62.78 + 13.64×A2H	0.23	1.38	<0.001
DE_1x_	Y = 3.09+0.66×Alpha	0.27	0.06	<0.001
DEp_3x_	Y = 3.03+0.41×Alpha	0.27	0.04	<0.001
ME_3x_	Y = 2.54+0.54×Alpha	0.27	0.05	<0.001
MEp_3x_	Y = 2.61+0.41×Alpha	0.27	0.04	<0.001
Nem_3x_	Y = 1.66+0.45×Alpha	0.25	0.04	<0.001
NEg_3x_	Y = 1.03+0.40×Alpha	0.26	0.04	<0.001
NELp_3x_	Y = 1.65+0.29×Alpha	0.25	0.03	<0.001
Estimated milk value (FMV)	Y = 2.46+0.44×Alpha	0.26	0.04	<0.001

FTIR, Fourier transform infrared; RSD, residual standard deviation; PA2, soluble true protein; PB1, moderately degradable protein; Total RDP, total rumen degradable protein; Total RUP, total rumen undegradable protein; Alpha, α-helix height; Beta, β-sheet height; Ratio, ratio of α-helix: β-sheet; AIIH, Amide II height; AI, Amide I area; Height, AIH:AIIH ratio; Area, AI:AII areas ratio; AECP, absorbed endogenous protein; ECP, endogenous protein; CP, crude protein; TDCP, total digestible crude protein; TDN, total digestible nutrients; DE, digestible energy; ME, metabolizable energy; NEm, net energy for maintenance; NEg_3x_, net energy for gain; NEL, net energy for lactation.

**Table 21:** Regression analysis to predict Canola carbohydrate parameters from FTIR carbohydrate structures

Variables (Y)	Prediction equation model: Y = a+b1×X1+b2×X2 …	R^2^	RSD	p-value
Canola meals				
CEL (% DM)	Y = 6.24+0.28×STCA	0.37	0.66	0.017
Total RDC	Y = 19.55+90.09×STC2H	0.35	1.01	0.021
MREE	Y = 52.46+1.61×STCA	0.34	4.04	0.022
Canola seeds				
HEMI (% DM)	Y = 15.72–0.58×STCA	0.30	1.30	0.034
DVE	Y = 37.28+129.46×TC4H	0.31	6.55	0.031
DVE FMV	Y = 0.76+2.62×TC4H	0.31	0.13	0.030

FTIR, Fourier transform infrared; RSD, residual standard deviation; CEL, cellulose; DM, dry matter; Total RDC, total rumen degradable carbohydrates; MREE, microbial protein synthesized in the rumen based on the energy available; HEMI, hemicellulose; DVE, truly digested protein in the small intestine; DVE FMV, estimated milk production based on the DVE system in kg milk/kg DM feed; STC, structural carbohydrate; TC, total carbohydrate; H, height; A, area; Numbers 2 and 4 correspond to different peaks.
